# Online flow cytometry, an interesting investigation process for monitoring lipid accumulation, dimorphism, and cells’ growth in the oleaginous yeast *Yarrowia lipolytica* JMY 775

**DOI:** 10.1186/s40643-016-0132-6

**Published:** 2017-01-03

**Authors:** Doria Naila Bouchedja, Sabine Danthine, Tambi Kar, Patrick Fickers, Abdelghani Boudjellal, Frank Delvigne

**Affiliations:** 1 Unité de Bio-Industries, Passage des déportés, Université de Liège/Gembloux AGRO-BIO Tech, 2-5030 Gembloux, Belgium; 2 INATAA, Université Frères Mentouri Constantine, Route de Aïn El Bey, 25000 Constantine, Algeria; 3 Unité de science des aliments et formulation Passage des déportés, Université de Liège/Gembloux AGRO-BIO Tech, 2-5030 Gembloux, Belgium

**Keywords:** *Yarrowia lipolytica*, Lipid accumulation, Cells’ shape, Cell growth, Online flow cytometry

## Abstract

This study aims to understand and better control the main biological mechanisms and parameters modulating the various phenomena affecting *Yarrowia lipolytica* JMY775 and its lipids accumulation. The results obtained in this study stress forward that the use of an original tool, consisting of coupling bioreactors to online flow cytometry, is highly efficient. Throughout 48 h of culturing, this emerging process allowed an online continuous observation of the effects of pH and/or aeration on the cell growth and dimorphism and lipid accumulation by *Y. lipolytica*. This present study showed clearly that online flow cytometry is an advantageous tool for the real-time monitoring of microbial culture at a single-cell level. Indeed, the present investigation showed for the first time that profiling of the various phenomena and their monitoring upon culture time is now possible by coupling online cytometry with culture bioreactors.

## Background

Less than 30 species of the 600 identified yeast species are known for their oleaginous property (Ratledge 2004). Thus, there is a great attention to the field of lipid production and metabolism from various unicellular organisms, particularly oleaginous yeasts which are suited to this production (Fickers et al. 2005). Furthermore, they are suitable for large-scale fermentation and are free of endotoxins. Hence, oils sourced from microbial cultures have similar lipid profiles in type and in composition to those produced by plants (Beopoulos et al. 2011). *Yarrowia lipolytica* is among the best used microbial cultures and the most interesting. In addition, thanks to its non-pathogenicity, spit of its dimorphic property which confers to it the ability to appear under a mycellar shape or yeast cell shape is the most studied non-conventional yeast. It has also the ability to store more than 20%, and even more than 40% for some strains, under particular conditions (Beopoulos et al. 2009).

Lipids are accumulated in *Y. lipolytica* by two antagonist ways: first, de novo biosynthesis of fatty acids, which is an anabolic pathway, corresponding to the production, under defined conditions, of fatty acids precursors, such as acetyl-CoA and malonyl-CoA, leads their integration in the lipids biosynthetic pathway (phospholipids, sphingolipids, triacylglycerol, etc). This pathway is so called the Kennedy pathway (Beopoulos et al. 2009; Papanikolaou and Aggelis 2011) and second, e*x* novo pathway corresponding to the incorporation of fatty acids, triglycerides, or alkanes, from the culture medium to the interior of the cell. This pathway requires the hydrolysis of the hydrophobic substrates, the transport and the incorporation of free fatty acids within the cell, and reassembly of stored fatty acids under triacylglycerides form, or sterol esters (Beopoulos et al. 2009). The accumulation and storage of fat are performed in specific compartments, called lipid bodies (Fujimoto et al. 2008).

Lipid accumulation in *Y. lipolytica* is influenced by several external factors, namely, dissolved oxygen, pH, presence of inorganic salts, and also temperature (Subramaniam et al. 2010). To rapidly determine physiological properties on single cell or to detect and identify the different sub-populations in a media, flow cytometry seems to be the appropriate tool for research on microorganisms. By this emerging tool, thousands of cells or molecules per second can be automatically analysed, thus making it a versatile and a fast technique even described as an automated microscopy (Ueckert et al. 1995; Díaz et al. 2010). Actually, several cellular parameters can be simultaneously measured, using modern laser-based flow cytometers. In flow cytometer when laser light beam hits a stream of cells or particles, it is scattered in different directions. This light is measured in forward scatter (FSC), whose detection can be viewed as an approximation of the detected cell or the particles and inside scatter (SSC) that is an approximation to granularity or shape (Bradner and Nevalainen 2003).

Thus, the objective of this study is to use for the first time flow cytometry for a better understanding and control of mechanisms and parameters modulating the different phenomena observed in *Y. lipolytica* culture. Mainly, this study allowed a fast investigation using this process, the growth dynamic, lipid accumulation, and cell dimorphism. The study of these phenomena and of the profile of their progress was possible thanks to the combination of the conventional analyses tools with flow cytometry as an-online culture monitoring.

## Methods

### Strain and mediums

The *Y. lipolytica* JMY 775 strain used in this study was obtained by genetic manipulation. It has an overproducing lipase strain (LgX64.81) modified with an *LIP2*-*LacZ* reporter gene (Kar et al. 2008). The yeast was relying on pre-cultivation in YPD medium for 24 h in 250 mL baffled shake flasks containing 100 mL of the medium at 30 °C, with vertical agitation (185 rpm). The pre-culture medium was composed of 5 g/L yeast extract (Organo Techni), 10 g/L peptone of casein (Organo techni), and as carbon source 20 g/L of glucose (Cargil, dextrose mono hydrate, 98% purity).

For the culture, 0.2 × 10^8^ cells were inoculated to 200 mL of lipid accumulator medium containing 5 g/L yeast extract, 5 g/L casein peptone, 10 g/L glucose, and 25 g/L oleic acid. After that, a twice sonication up to 85% for 120 s was performed to emulsify the medium.

### Bioreactor operating conditions

The yeast culture was carried out in continuous mode, during 48 h in three multiphasic complex mini bioreactors (Dasgip 4Unit Technologie), mechanically stirred, with a working volume of 200 mL. The used bioreactors worked in parallel and were connected to a data control computerized system (DasgipAc 4.5). Each bioreactor was fitted with sensors of pH, pO_2_, DO (dissolved oxygen), and pressure. The operating temperature was fixed at 28 ± 0.2 °C. For the control bioreactor (named F1), pH was adjusted to 6.0 ± 0.05 and DO to 800 rpm at 5pV. For the pH bioreactor (named F2), pH was dropped and not adjusted. For the dissolved oxygen bioreactor (named F3), the dissolved oxygen amount was less than in the two other bioreactors, inducing an oxygen limitation of 300 rpm at 5pV. These adjustments make possible the observation of the effect of each parameter on: (1) cell growth; (2) lipid accumulation; and (3) cells’ shape changes.

### Optic and fluorescence microscopy

The cells’ growth rating on Bürker cell and lipid accumulation revealed by Nile-red fluorescent dye (Sigma-Aldrich, N3013) was performed using optic and red fluorescence microscopy (microscope Zeis-Axioscop 2MOT), respectively. The microscope was coupled to a camera-type Axiocam HRC color Carl Zeis technology, operating with Axiovision logiciel version 3.1. The staining technique was as described by Beopoulos et al. (2008) allowing the observation by fluorescence microscopy of lipids accumulated by *Y. lipolytica* during culture.

### Spectrophotometry

The quantitation of extracellular oleic acid remaining in the culture medium has been performed using spectrophotometric measurement as described by Izard and Limberger (2003).

### Glucometry

The quantitation of total glucose in the culture medium allowed the estimation of cells’ glucose consumption during the culture and was performed using a glucometer (YSI, 2700-D Biochem). The samples were analysed after different dilutions (0×, 10× and 100×) and monitored at different experiments times (6, 24, 30, and 48 h).

### Online flow cytometry

The bioreactors were online coupled as indicated above to a flow cytometer (Accuri C6 flow cytometer). This coupling process was developed for the first time in this study to observe online and in real-time the most phenomena occurring over the culture (cell growth dynamics, cells’ shape changes, cells’ fluorescence, and lipid assimilation) according to the culture factors in each of the bioreactors. An interface allowed an automated sampling every 60 min. The samples were systematically diluted with distilled water to obtain 40,000 events per sample thus avoid overlap between event and signal distortion induced by high sample concentrations. Overall, flow cytometer measure quantitatively the optical characteristics of cells or particles (also called events) presented in a single file in front of a focused light beam. As particles filter through this latter, three parameters were measured using photomultiplier tubes. They are forward scatter (FSC), side scatter (SSC), and fluorescence (FL). The cell sizes were inversely proportional to the amount of light scattered forward and at right angles. Furthermore, the cell refractibility is related to surface properties and internal structure affecting FSC and SSC. The fluorescence was measured in three wavelength ranges. The light-defined wavelength is channelled to particular and detectors; these are specific for each fluorescence range. Thus, green fluorescence is typically measured by FL1 detector, whereas orange fluorescence and red fluorescence were, respectively, measured by FL2 and FL3 detectors.

## Results

### Cell growth and lipid assimilation revealed by online flow cytometry

**Fig. 1:**
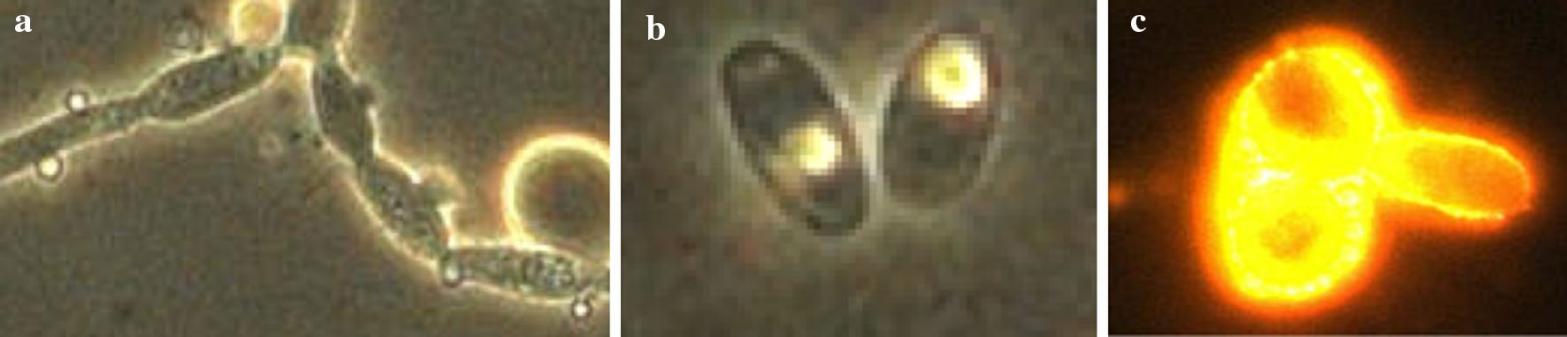
**a** Optic microscopy images (100×) after 48 h of culturing, showing the presence of protrusions on surface of *Y. lipolytica* cell. Yeast culture was carried out in bioreactor containing YPD medium enriched with oleic acid (YPDOA), at 28 °C, pH 6.0 and dissolved oxygen limitation. **b** Optic microscopy image (100×) showing lipid bodies within *Y. lipolytica* cells, after 24 h of culturing in YPDOA. Culture was carried out in bioreactor, at 28 °C, pH 6.0, without dissolved oxygen limitation. **c** Fluorescence microscopy image (100×) showing high intracellular lipid accumulation in a cell of *Y. lipolytica.* Intracellular lipids were revealed with *Nile-red* staining; as well intracellular assimilated lipid displayed a red fluorescence. Sampling was carried out after 30 h of culturing in bioreactor at 28 °C, pH 6

**Fig. 2:**
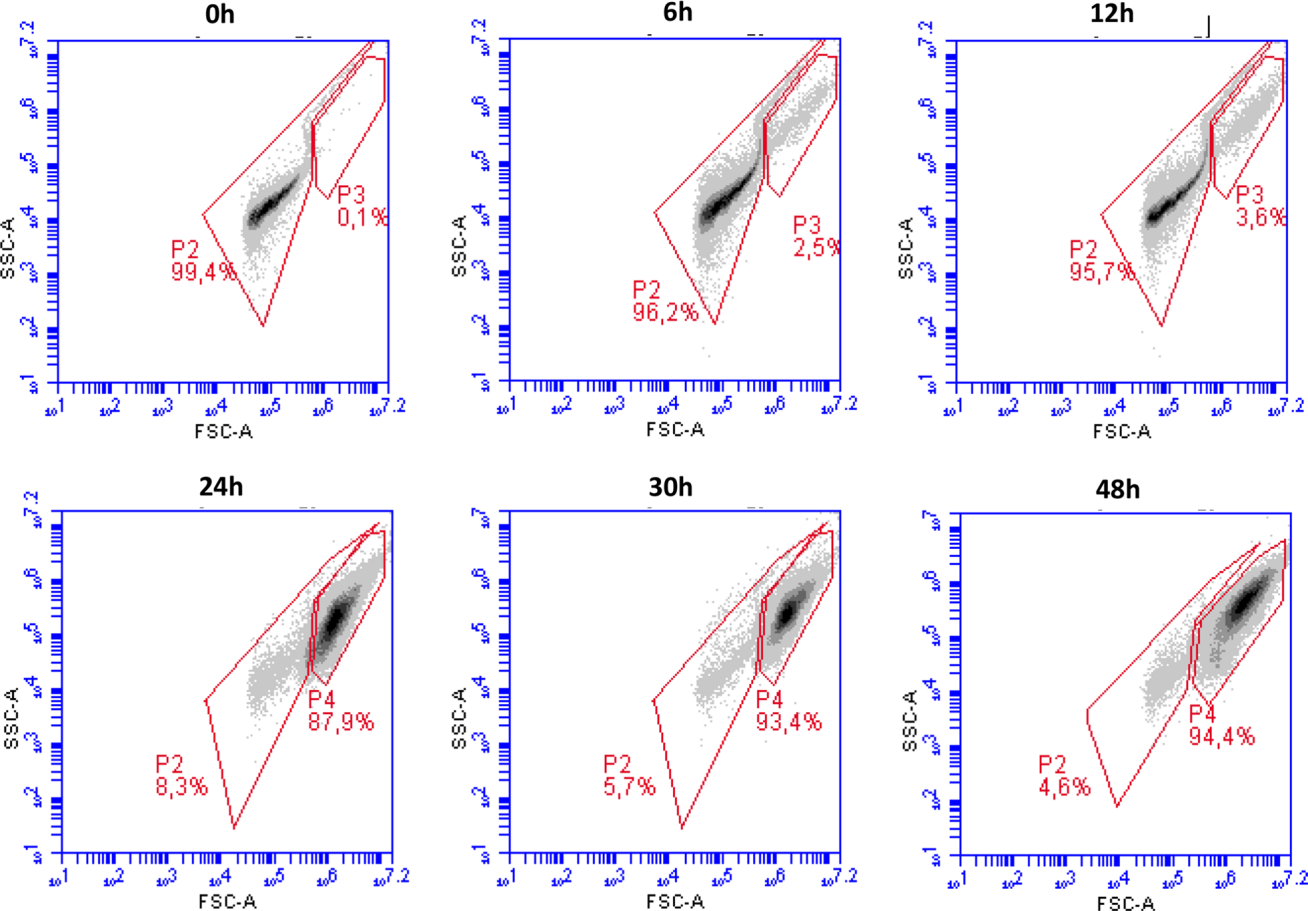
Evolution of the oil micro-droplets (gate *P2*) and yeast cell population (gates *P3*, *P4*) during a culture of *Y. lipolytica* JMY 775 carried out in a single stirred mini-bioreactor. Analyses have been carried out with online FC and some of the results are displayed on forward scatter/side scatter (FSC/SSC) dot-plot on the basis of 40,000 events

**Fig. 3:**
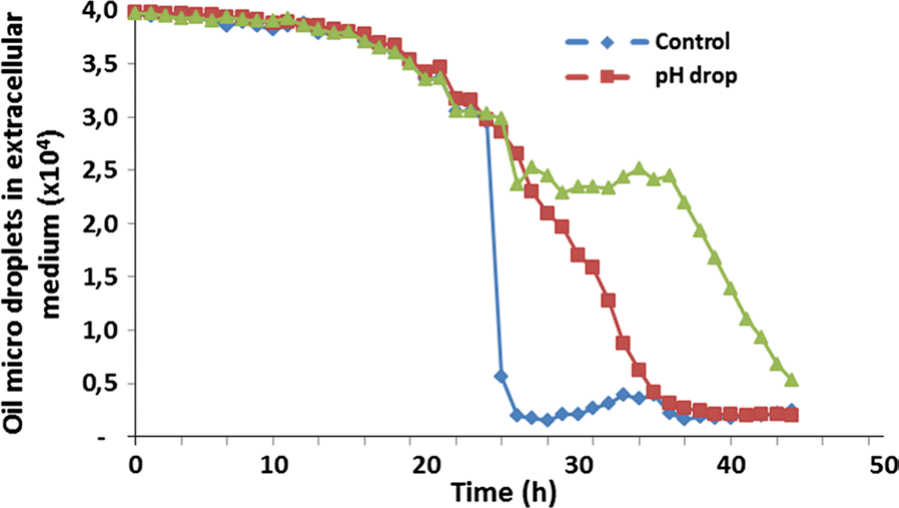
Evolution of oil droplet fraction in the function of different bioreactor operating conditions. Bioreactors have been sampled by online flow cytometry. Percentage of oil droplets has been determined by gating individually microbial cells and oil droplets according to the procedure displayed in Fig. 3

Nile-red-stained cells growing on enriched medium with oleic acid (OA) showed small protrusions scattered across their surfaces (Fig. 1). The hydrophobic substances assimilation by *Y. lipolytica* showed that it requires morphological and physiological adaptations. Thus, lipid droplets on cells’ surface could be observed once the protrusion number increased as can be shown in Fig. 1. The demarcation of cell growth and oil micro-droplets dot-plot areas on the basis of FSF/SSC parameters allowed a clear observation the evolution of cells’ growth and cells’ lipid assimilation. Thus, cytograms revealed that throughout culturing, cells’ growth dot-plot areas were continually expanding, whereas medium’s oleic acid was absorbed by cells (Fig. 2 and see also Fig. 3).

### Cells’ shape changes revealed by online flow cytometry

**Fig. 4:**
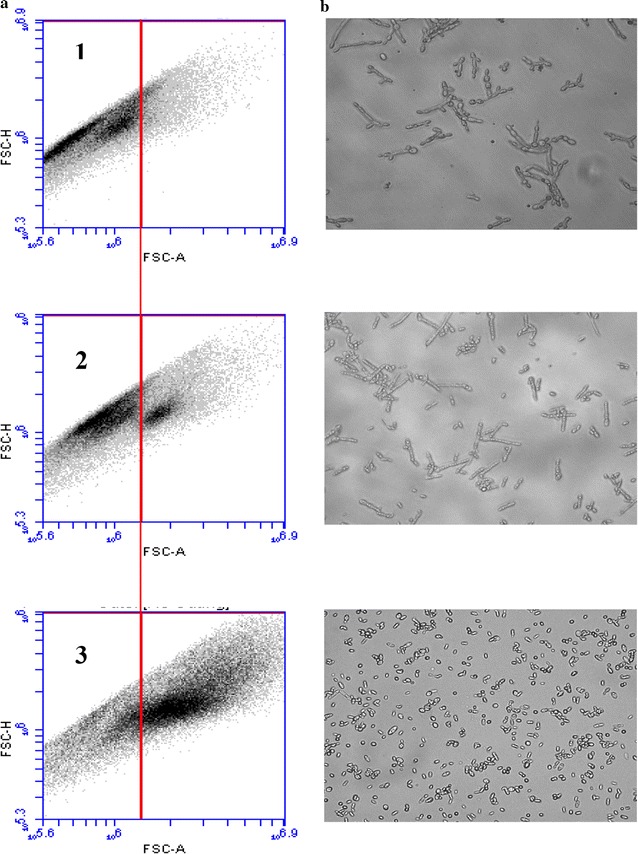
Cell dimorphism reviled by online flow cytometry and optic microscopy. **a** Cell dimorphism occurring during a culture of *Y. lipolytica* JMY 775 in bioreactor and detected with online flow cytometric process. After gating two sub-populations, different morphologies can be distinguished, i.e., *a1* elongated shape cell sub-population, *a2* both morphotypes can co-exist in the same culture, and *a3* single-cell shape sub-population. Analyses have been carried out with online FC and results were displayed on Forward Scatter Air/Forward Scatter Height (FSC-A/FSC-H) dot-plot on the basis of 40,000 events. **b** Optic microscopy images (40×), corresponding to samples of the panel “**a**”

By the same process, on basis of FSC-Air/FSC-height parameters, the demarcation of dot-plots corresponding to different cells’ morphologies was also possible. Thus, with this tool, the different cells’ shapes have been distinguished on the obtained cytograms. Once again, standards were used for locating of each different shape’s area (Fig. 4). It is worth mentioning that *Y. lipolytica* has the ability to appear in different shapes, i.e, elongated shape or single-cell shape. In some cases, both forms co-existed within the same culture for the same strain, as can be seen in Fig. 4a2 and b2. These morphological cell changes often occur under external factors which were studied easily and followed at each moment of the culture with online flow cytometry.

### Monitoring of culture parameters effect on growth dynamics and on lipid accumulation

**Fig. 5:**
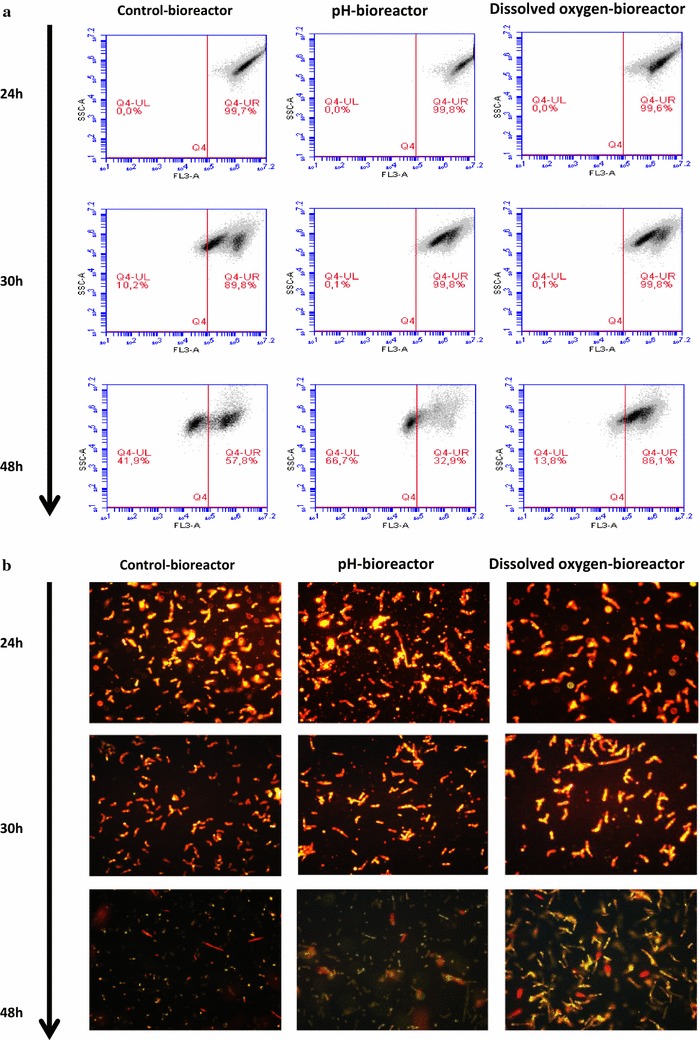
Monitoring culture parameter effect on lipid accumulation in *Y. lipolytica* JMY775 reviled by online flow cytometry and fluorescent microscopy analyses. **a** Results corresponding to the samples displayed in panel **b** (FL3-A: red fluorescence intensity proportional to *Nile-red* staining; SSC-A: side scatter intensity). **b** Microscopy images (40×) showing the lipid accumulation in the yeast for different bioreactor operating conditions (control bioreactor: culture operated with dissolved oxygen and pH controls; dissolved oxygen bioreactor: culture operated with dissolved oxygen limitation; and pH bioreactor: bioreactor operated without pH control). For each condition, cells have been sampled at different time intervals and stained with *Nile-red*

The demarcation of dot-plots corresponding to different levels of red fluorescence was also possible using online flow cytometry on basis of FL3-Air/SSC–Air parameters (Fig. 5a). The intensity of red fluorescence within culture was proportional to the degree of cells’ lipid assimilation and/or lipid accumulation (Fig. 5a, b).

**Fig. 6:**
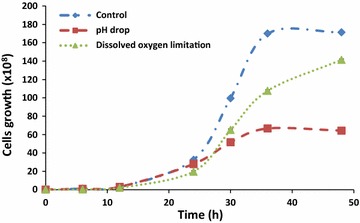
Cells’ growth profiles during 48 h of *Y. lipolytica* JMY 775 culturing in bioreactors, under different conditions of pH and aeration

**Fig. 7:**
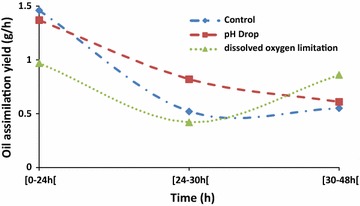
Oleic acid assimilation yield during 48 h of culturing under different conditions of pH and aeration

**Fig. 8:**
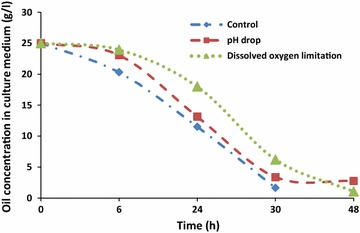
Extracellular oleic acid concentration during 48 h of culturing in bioreactors, under different conditions of pH and aeration

**Fig. 9:**
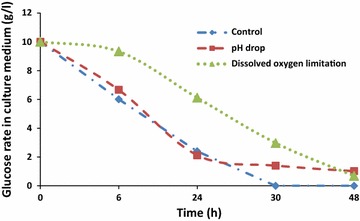
Glucose medium concentration during 48 h of culturing in bioreactors, under different conditions of pH and aeration

**Fig. 10:**
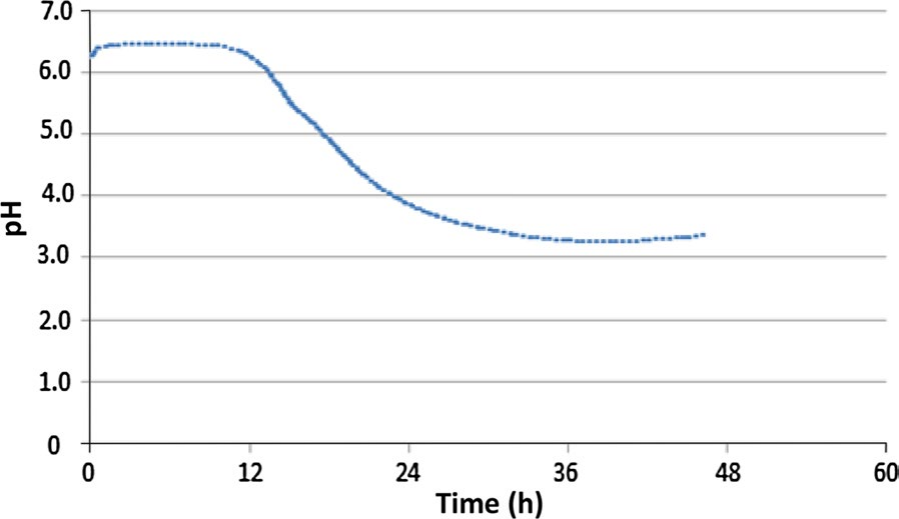
pH changes in the unregulated-pH bioreactor (F2), during 48 h of *Y. lipolytica* JMY 775 culturing

At the end of culturing, almost oil present in the medium was assimilated in each bioreactor (Fig. 8). However, it seems that these phenomena occur by different ways depending on the culture conditions. Thus, at the beginning of a culture, the amount of oleic acid varied slowly in each bioreactor (Fig. 6). The same trend was also observed concerning cell growth which starts an exponential increasing until 24 h of culture. After 10 h of culture, the increase of the biomass was accompanied by a rapid assimilation of oleic acid. In addition, this was fewer the F3 bioreactor. In control bioreactor (F1), oleic acid attained the lowest level at an average of 30 h of culturing (Fig. 7). Nevertheless, with the combination of cytometry results and those of usual techniques, it could be clearly seen that in all reactors, the step corresponding to rapid medium’s fat absorption coincides with the phase of glucose depletion (Figs. 6, 7). Though, due to the optimized culturing conditions, cells’ growth and fat’s absorption appear faster in control bioreactor (F1). In F3 bioreactor, the rate of cells’ oil assimilation was slower in comparison with the two other bioreactors (Fig. 6). In those conditions and at the beginning of culture, cells’ growth and lipid accumulation rates were lagged (oxygenation was the lowest), but they were more fluorescent at the end of the experiments than the cells of the other bioreactors. Therefore, they appeared still in growth (Fig. 6), whereas glucose was completely exhausted in the medium (Fig. 9). It seems also that under conditions of the not adjusted pH (pH-drop bioreactor: F2), oleic acid assimilation decreased when pH decreased at a value of 4.0 (Figs. 7, 10). Thus, after 48 h of culturing, cells in F2 appear less fluorescent, whereas oleic acid still remaining in the culture medium (Figs. 5, 8). The same trend was observed with glucose absorption and cell growth; which were slowed in F2 when pH attained the critical pH value of 4.0. Even though medium’s glucose was not totally consumed (Figs. 9, 10).

### Monitoring of culture parameter effect on cells’ shape changes

**Fig. 11:**
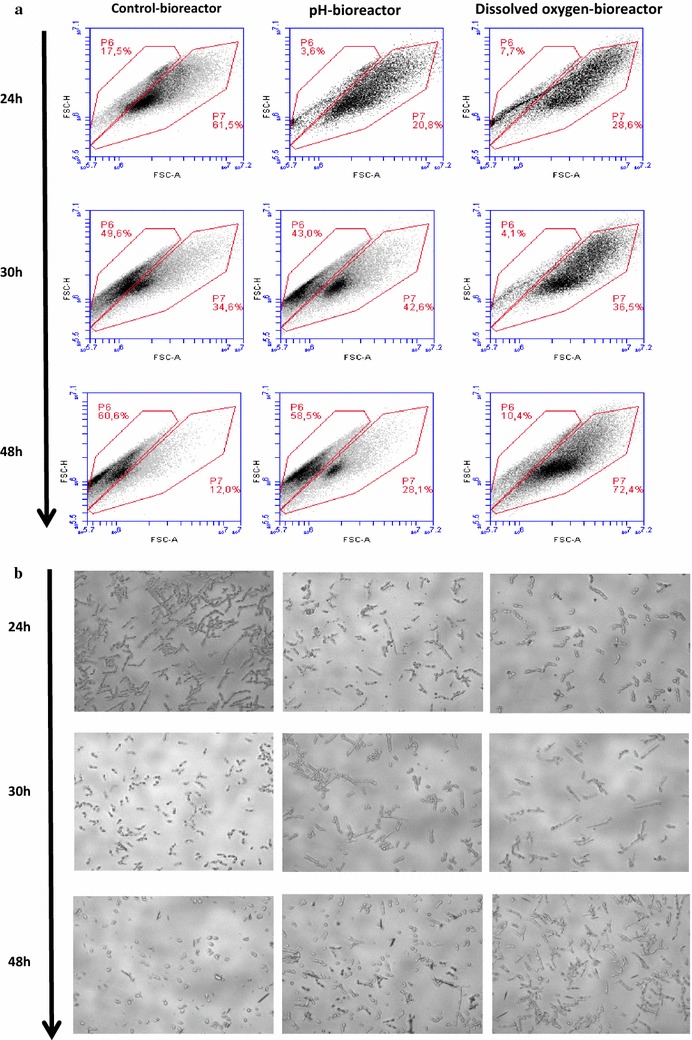
Monitoring culture parameter effect on cell shape changes reviled by online flow cytometry and optic microscopy. **a** Flow cytometry analyses corresponding to the samples displayed in panel B, reviling different morphotypic sub-populations in each bioreactor (results were displayed on Forward Scatter Air/Forward Scatter Height (FSC-A/FSC-H) dot-plot on the basis of 40,000 events). **b** Optic microscopy images (40×) showing the yeast *Y. lipolytica* JMY775 under different shapes for different bioreactor operating conditions (control bioreactor: optimized culture operated with optimized dissolved oxygen and pH controls; dissolved oxygen limitation-bioreactor: culture operated with dissolved oxygen limitation and controlled pH; and pH-drop bioreactor: culture operated without pH control but also without oxygen limitation). For each condition, cells have been sampled at different time intervals

At the beginning of the experiments, cytograms revealed homogeneous populations within each bioreactor. The microscopic graphs taken at the end of the experiments (48 h) enabled to observe that in the F1 bioreactor (optimized conditions), the cells took a yeast-like form. In the F2 bioreactor, a transitional situation was observed, in which co-inhabited both mycelar and yeast-like forms. Finally, F3 bioreactor cells at the end of culturing appeared more voluminous with elongated and homogeneous forms (Fig. 11).

## Discussion

*Yarrowia lipolytica* is able to use free fatty acids or to store them as triacylglycerol’s (TAG) and stearyl esters (STE) into lipid bodies depending on the environmental conditions. A lipid body is a hydrophobic core formed from neutral lipids, mainly TAG and in a lesser amount as STE (Papanikolaou et al. 2006; Beopoulos et al. 2009; Nicaud 2012). As described in earlier studies, a mixture of hydrophilic and hydrophobic substrates had been used, i.e, glucose and oleic acid. Hydrophobic substance assimilation by *Y. lipolytica* requires usually morphological and physiological adaptations. For example, Mlíčková et al. (2004) found that cells growing on glucose had smooth surfaces. In agreement to this present study, cells growing on a medium enriched with fatty acids had small protrusions scattered across their surfaces, especially those which had taken a mycelar form. The number of protrusions increased with the time of incubation in oleic acid, so at the beginning of the experimentation, protrusion number increased from 48 h after the induction. The lipid droplets on cells’ surfaces were also observed and their number increased when protrusion were several. These findings are in agreement with the observations reported in numerous previous studies (Mlíčková et al. 2004; Fickers et al. 2005; Beopoulos et al. 2008, 2009; Coelho et al. 2010).

The findings showed that in all the bioreactors, the step corresponding to a fast medium’s fat consumption coincides with the step of glucose depletion. (Fickers et al. 2005) reported that few lipid bodies could be observed in *Y. lipolytica* when grown in glucose medium, whereas lipid body accumulation was observed during culture in fatty acid or triglyceride medium. This suggests that when growing on a mixed medium composed of sufficient quantities of glucose and oleic acid, yeast cells began to metabolize first glucose as preferential carbon source (Papanikolaou et al. 2006; Weinhandl et al. 2014). After that, when glucose decreases greatly, cell fatty acid accumulation was triggered. This is clearly shown by cells’ growth and oleic acid accumulation which slowed after 30 h of culturing, both in control (F1) and in pH-drop bioreactor (F3). Nevertheless, growth and lipid accumulation appeared to extend in F3. Thus, we can suggest that stationary phase may occur probably when cells metabolized by the oxidation of their intracellular accumulated lipids (using them as carbon source instead of glucose). This process was independent of the nitrogen concentration in the culture medium as already reported by Papanikolaou et al. (2002). However, low oxygenation rate in F3 enabled to slow oxidation of carbon sources, and leads to extend the time of intracellular lipid accumulation and cell growth phase. Previous studies showed that the improvement of the oxygen availability either by acting on saturation level (5–15%) (Papanikolaou et al. 2002), on air pressure (6 bar) (Lopes et al. 2009), or on the aeration inside the bioreactor (Coelho et al. 2010) and have a significant effect in cells’ growth rate. Thus, as oxygenation is a very important parameter, it is useful to know how to manage this parameter in a bioprocess. In this context, Coelho et al. (2010) reported that oxygen transfer rate (OTR) allows the analysis of the oxygen impact on a bioprocess. For a specific bioreactor and medium, OTR could be increased by increasing agitation and aeration rate (Coelho et al. 2010). This statement was observed in this study. Furthermore, an increase of lipase production and lipolysis activity in *Y. lipolytica* was previously described (Alonso et al. 2005; Coelho et al. 2010). We suggest that these may be verified by reducing agitation in the F3 bioreactor.

The developed online flow cytometry process allowed an efficient monitoring and in a real-time manner of the lipid accumulation during the primary anabolic growth phases under the influencing medium’s pH, incubation temperature, and aeration parameters. Accordingly, Papanikolaou et al. (2002) reported that at pH 6.0 and a temperature of 28 °C, high quantities of dry biomass may be produced in shorter time, whereas significant quantities of lipids substrate were rapidly consumed by yeast cells. Indeed, under optimal conditions of aeration (600 rpm), temperature (28 °C), and pH 6.0, cells’ growth was faster in F1 bioreactor. This may be explained by the fast consumption of both glucose and oleic acid, as carbon sources by the cells. These findings are supported by the results previously reported by Papanikolaou et al. (2002).

pH and incubation temperature have been reported as significant factors. They are in relation with the process of single-cell oil accumulation. For example, the influence of pH was already studied when *Y. lipolytica* was growing on stearin (10 g/L) in the presence of (NH_4_)_2_SO_4_ and at 28 °C. Thus, it seems that pH has a crucial effect on single-cell oil accumulation, and also on cells’ growth. Therefore, in their investigation related to the effect of medium pH on lipid production by *Rhodosporidium toruloides* strain, (Dias et al. 2016) found that yeast biomass concentration and lipid amount were the highest at pH 4.0. Using the same operating process, we observed that fat accumulation by *Y. lipolitica* JMY755 was more efficient at pH 6.0 in contrast to the results by Papanikolaou et al. (2002) reporting that at pH 5.0 and 7.0, the cell growth was narrowed. Therefore, in this report, under the unregulated-pH conditions (F2), the consumption of fatty acids was slowed when pH decreased under the value of 5.0. Thus, after 48 h of culturing, cells in F2 looked barely fluorescent and medium’s oil rate starts dropping as in F1, but with a lower rate. The minimal level of medium fatty acids was attainted in F2 after 36 h of culturing. This may be probably caused by the important pH drop due to the critical pH value (pH ≤4.0) at 24 h of culturing. Thus, under critical pH value, glucose consumption was completely inhibited, while intracellular lipid assimilation was affected by the significant decreasing of pH, hence, affecting well cell growth. The optimization of culture parameters allowed a faster cell growth process and energetic metabolism. In these conditions, glucose as a preferred energy source was rapidly metabolized in the oxidation process, supported by better availability of oxygen (in both F1 and F2). Thus, glucose was rapidly used by the biomass and the oleic acid consumption started to operate as soon as glucose was used. Several experiments, in which fatty acids were added into the growth medium, showed an important fatty acid biosynthesis through de novo mechanism especially when cells assimilated high sugar quantities (Papanikolaou et al. 2006). A decrease of lipid storage in *Y. lipolytica* occurs when growth was done on low substrate fat concentration media. Oleaginous microorganisms growing on fats commonly consume their own storage lipids when the flow rate of exocellular fatty acids is considerably decreased in the culture medium (Aggelis et al. 1995). Lipid accumulation from fats was reported as a result of an unbalanced uptake and assimilation rate of aliphatic chains. When this uptake is higher than that of the assimilation rate, lipid is accumulated inside of the microbial cell. In contrary, when the assimilation rate is higher than the substrate fat, the growth needs are covered by the degradation of storage lipid (Papanikolaou and Aggelis 2003). These findings were verified in our study using online cytometric process. In addition, this process allowed to observe that at the end of culture, cells derived from control bioreactor (F1) were not fluorescent; in contrast to those from F3. It seems that, in limited-oxygen bioreactor (F3), cells still fluorescent after 48 h of culturing.

The present study showed also that different parameters may play significant roles in *Y. lipolytica* dimorphism. For example, at 24 h of culturing, almost of the cells presented a pseudo-mycelial form with no yeast-like form. Since the 1990s, the undertaken dimorphism studies on *Y. lipolytica*, showed its ability to grow as a yeast-like or hyphal forms, depending on growth conditions (Fickers et al. 2005). Furthermore, *Y. lipolytica* was reported as a model for dimorphism (Barth and Gaillardin 1997; Nicaud 2012). Thus, the cytograms of this study revealed clearly homogeneous populations within each bioreactor. Microscopic observations enable to note that at the end of culturing (48 h), cells in the F1 took a yeast-like form, while in the F2, a transitional situation was observed. However, in F3, cells appeared still filamentous with elongated and homogeneous forms but more voluminous than at the beginning of experiment. (Makri et al. 2010) reported that during biomass production, phase short and true mycelia and pseudo-mycelia may predominate. Indeed, large obese cells with discernible lipid globules appeared in the early stationary phase, namely, in the lipogenic phase. At this phase, the lipid globule sizes were diminished during the late stationary phase, namely, at the citric acid production phase (Makri et al. 2010). This phase appeared earlier in F1 and F2 bioreactors than in F3. Moreover, it has been reported that during the exponential growth phase, when mycelial form dominated over yeast form, high respiration activity may be observed (Makri et al. 2010). A gradual transition to yeast-like cells form in lipogenic and in citric acid production phases appeared in the F2 bioreactor. The decline of pH may explain part of these findings.

On another hand, cell size was affected by the different percentages of the accumulated lipids among lipogenic and citric acid production phase as reported by Makri et al. (2010). However, many inconsistencies were reported by earlier studies about *Y. lipolytica’s* dimorphism. For example, (Rodriguez and Domínguez 1984) reported that elongation may operate only upon entering the stationary phase, and during the growth phase, *Y. lipolytica* may stay in yeast-like mode (Chen et al. 1997; Makri et al. 2010). The differences may be explained by the effect of temperature (Makri et al. 2010) due to heat shock (Guevara-Olvera et al. 1993), to the atmosphere composition and in the presence of specific compounds in the culture media (Barth and Gaillardin 1997; Cervantes-Chávez et al. 2009), as well as the nature of carbon substrate (Rodriguez and Domínguez 1984; Guevara-Olvera et al. 1993; Ruiz-Herrera and Sentandreu 2002), and to the atmosphere of growth media and pH (Ruiz-Herrera and Sentandreu 2002). Other studies (Barth and Gaillardin 1997; Zinjarde et al. 1998) also suggest that the capacity of dimorphism is strain specific. In this context, (Szabo and Štofaníkova 2002) confirmed that external pH regulates dimorphism predominantly via modulation of the availability and/or the use of the organic sources of nitrogen in *Y. lipolytica*. Though, we could be confronted once again to some inconsistencies to some of the reported studies concerning the effect of this parameter. Therefore, when some of the studies conclude that mycelium formation was maximal at pH near neutrality and decreased as pH was lowered to converge to zero at pH 3.0 (Ruiz-Herrera and Sentandreu 2002), other studies reported that under particular conditions, mycelial form may be observed on cells subjected to pH shock (Kar et al. 2011) or on cells exposed to acidic pH (Szabo 1999).

## Conclusion

Online flow cytometry process appears as an efficient tool for monitoring a bioreactor culture of *Y. lipolytica*. This study showed the great potential of this emerging and versatile technique, not only for the study of the involved parameters cell culture evolution, but also into study of the various phenomena induced by modifications in each culture condition. Thus, it was proposed to offer a considerable time saving for obtaining and processing results in comparison with the conventional techniques.
